# Compound Heterozygous Protein C Deficiency Presenting With Splenic Infarction After COVID-19: A Case Report

**DOI:** 10.7759/cureus.101852

**Published:** 2026-01-19

**Authors:** Saki Imai, Soichiro Ishimaru, Masayuki Hirai, Makito Tanaka, Tetsushi Yoshikawa

**Affiliations:** 1 Department of Pediatrics, Kariya Toyota General Hospital, Kariya, JPN; 2 Department of Pediatrics, Fujita Health University School of Medicine, Toyoake, JPN

**Keywords:** anticoagulation therapy, covid-19, protein c deficiency, splenic infarction, venous thromboembolism (vte)

## Abstract

Congenital protein C deficiency is a rare autosomal recessive disorder that predisposes patients to severe thrombosis due to markedly reduced protein C activity. Although primarily associated with venous events, an increased risk of arterial thrombosis is also recognized, particularly in severe cases. We report the case of a 17-year-old male patient with compound heterozygous protein C deficiency who developed venous thromboembolism (VTE) after coronavirus disease 2019 (COVID-19) and subsequently experienced splenic infarction despite anticoagulant therapy. Diagnosed shortly after birth following neonatal purpura fulminans, he had been maintained on long-term warfarin therapy. The patient presented with fever and cough and was diagnosed with COVID-19 by antigen testing. Soon after symptom onset, he developed right thigh pain with rapidly expanding ecchymosis, leading to a diagnosis of recurrent VTE. Intravenous heparin and fresh frozen plasma (FFP) were initiated. Although his symptoms and coagulation markers improved by day 10, he developed sudden abdominal pain on day 11, and a contrast-enhanced CT scan revealed a partial splenic infarction. Anticoagulation was intensified, and he subsequently recovered without sequelae. COVID-19 can precipitate both venous and arterial thrombosis through endothelial injury and inflammation. This case illustrates that thrombotic events may occur even after apparent clinical and biochemical improvement, emphasizing the need for careful monitoring and individualized anticoagulation strategies in patients with protein C deficiency during and after COVID-19.

## Introduction

Congenital protein C deficiency is a rare autosomal recessive disorder that leads to severe thrombotic events due to markedly reduced protein C activity, with an estimated incidence of one in 500,000-750,000 live births among homozygous or compound heterozygous patients [1]. Coronavirus disease 2019 (COVID-19) causes vascular endothelial injury and systemic inflammation, increasing the risk of venous and arterial thrombosis [2,3]. Because protein C also exerts endothelial-protective effects, its reduced activity may further amplify COVID-19-associated coagulopathy [4,5]. There is, however, only limited evidence to describe how congenital protein C deficiency interacts with post-COVID vascular endothelial dysfunction, particularly regarding arterial thrombosis in the recovery phase.

Here, we report the case of a patient with compound heterozygous protein C deficiency who developed venous thromboembolism (VTE) after COVID-19 and subsequently experienced splenic infarction despite apparent biochemical improvement and tapering of anticoagulation.

## Case presentation

A 17-year-old male patient presented with fever and cough and was diagnosed with COVID-19 by antigen testing two days after symptom onset.

The patient had been followed for compound heterozygous protein C deficiency at our hospital since birth. He presented with purpura fulminans shortly after birth and was diagnosed with protein C deficiency (protein C activity <10%, protein C antigen <5%), maintaining a prothrombin time-international normalized ratio (PT-INR) of approximately 4. Genetic analysis had revealed compound heterozygous mutations in the protein C gene (*PROC*), the inactivator of coagulation factors Va and VIIIa (c.631C>T and c.1268delG), inherited from each parent. These variants have been previously reported in Japanese patients with severe congenital protein C deficiency [6]. The patient had recurrent episodes of VTE triggered by infection or trauma and had been treated with heparin and periodic fresh frozen plasma (FFP) supplementation.

At the current presentation, he had no history of COVID-19 vaccination and had not received antiviral therapy. The warfarin dose was increased from 6.0 to 6.5 mg/day because his PT-INR had decreased during the early phase of COVID-19 infection. Shortly thereafter, he developed right thigh pain with rapidly expanding ecchymosis and was admitted the same day for evaluation and treatment of suspected VTE. On admission (defined as day 1), laboratory tests showed elevated D-dimer (1.7 μg/mL) and thrombin-antithrombin complex (TAT) (7.0 ng/mL), consistent with VTE (Table 1).

**Table 1:** Representative laboratory findings on hospital day 1 Neut, neutrophils; Lym, lymphocytes; Alb, albumin; T-bil, total bilirubin; Cr, creatinine; TP, total protein; AMY, amylase; GLU, glucose; CRP, C-reactive protein; PIC, plasmin-α2-plasmin inhibitor complex; TAT, thrombin-antithrombin complex; PAI-1, plasminogen activator inhibitor-1; WBC, white blood cells; RBC, red blood cells; Hb, hemoglobin; Hct, hematocrit; Plt, platelets; PT-INR, prothrombin time-international normalized ratio; APTT, activated partial thromboplastin time; BUN, blood urea nitrogen; AST, aspartate aminotransferase; ALT, alanine transaminase; LDH, lactate dehydrogenase

Parameters	Patient Value	Reference Range
WBC (/µL)	5900	4000–11000
Neut (%)	52.2	40–70
Lym (%)	29.1	20–50
RBC (×10⁶/µL)	4.98	4.3–5.7
Hb (g/dL)	12.4	13–18
Hct (%)	40.1	40–52
Plt (×10⁵/µL)	3.06	1.5–4.0
PT-INR	2.13	0.9–1.1
APTT (s)	53	25–35
D-dimer (µg/mL)	1.7	<1.0
TAT (ng/mL)	7	0–4
PIC (µg/mL)	0.76	0.2–0.8
PAI-1 (ng/mL)	20.6	5–40
Sodium (mEq/L)	141	135–145
Potassium (mEq/L)	4	3.5–5.0
Chloride (mEq/L)	106	98–108
BUN (mg/dL)	15.7	7–20
Cr (mg/dL)	0.55	0.5–1.1
TP (g/dL)	7.1	6.5–8.0
Alb (g/dL)	4.2	3.5–5.2
T-bil (mg/dL)	0.2	0.2–1.2
AST (U/L)	20	13–33
ALT (U/L)	16	8–42
LDH (U/L)	225	120–245
AMY (U/L)	66	40–120
GLU (mg/dL)	105	70–110
CRP (mg/dL)	0.17	<0.3

Given his history of multiple VTE episodes with identical presentations, recurrent VTE was clinically diagnosed based on physical findings and corroborative laboratory data without additional imaging at that time, to expedite treatment initiation. Continuous intravenous unfractionated heparin at 18 units/kg/hour and FFP at 16 units/day were immediately initiated, while warfarin was continued throughout the treatment period, in accordance with his long-term management protocol for severe protein C deficiency.

**Figure 1:**
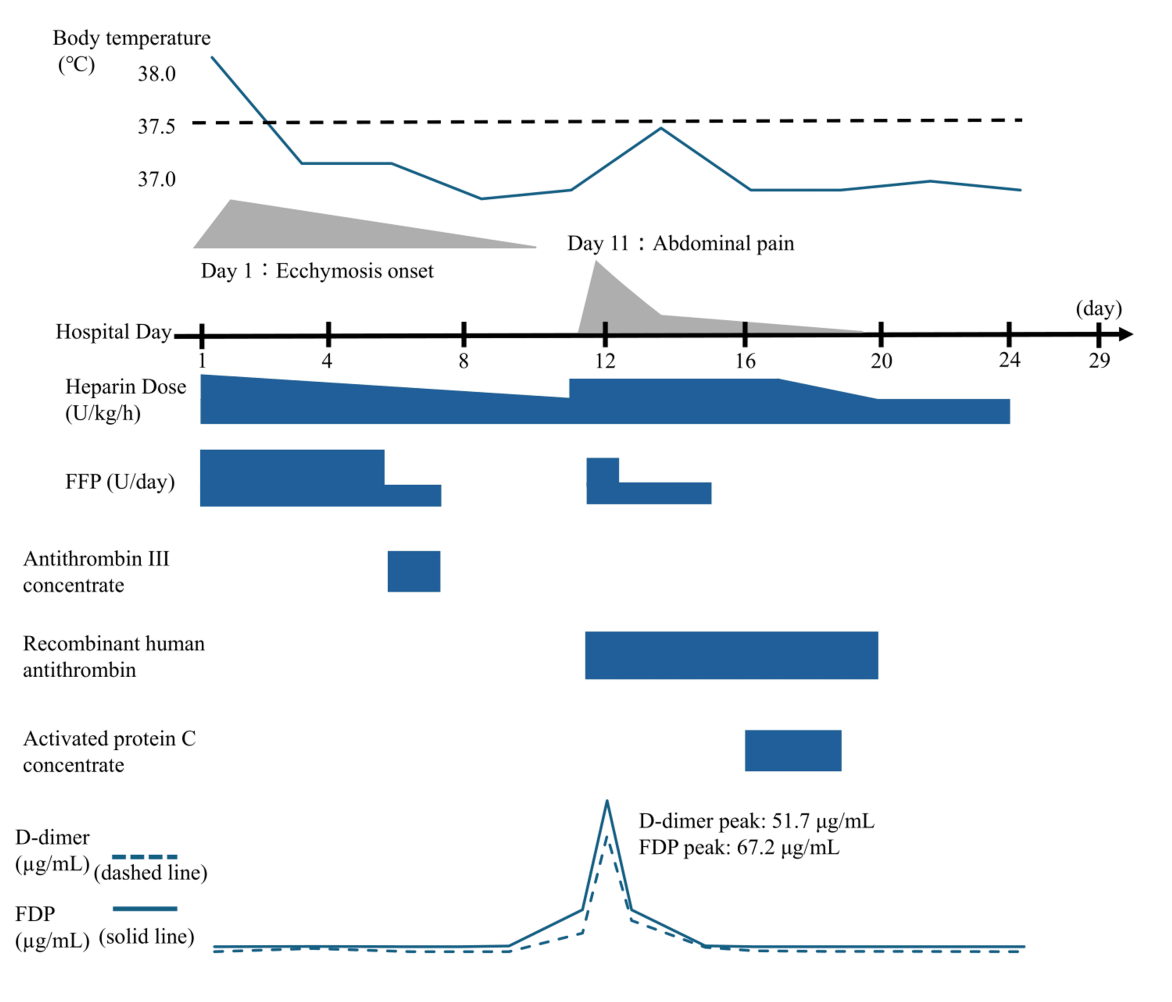
Clinical course during hospitalization Trends in body temperature, D-dimer, and fibrin degradation products (FDP) levels are shown, along with the progression of right thigh ecchymosis and the onset of abdominal pain. The timeline displays hospital days along with the corresponding doses of unfractionated heparin (U/kg/h) and daily fresh frozen plasma (FFP) administration (U/day). Ecchymosis gradually improved following the initiation of anticoagulation therapy. Abdominal pain emerged on hospital day 11, coinciding with a sharp rise in coagulation biomarkers and the diagnosis of splenic infarction. Adjustments in heparin and FFP dosing, including temporary discontinuation of FFP on day 8 and reinitiation from days 12 to 15, illustrate the therapeutic decision-making throughout the hospital course. Antithrombin III concentrate (Nonthron; 1,500 units/day for two days), recombinant human antithrombin (Acoalan; 1,800 units/day from days 11 to 20), and activated protein C concentrate (Anact C; administered for three days) are also indicated on the timeline.

Antithrombin supplementation with antithrombin III concentrate (Nonthron; Takeda Pharmaceutical Co., Ltd., Tokyo, Japan) at 1,500 units/day was initiated on day 6 and continued for two days. Heparin and FFP were gradually tapered as shown in Figure 1 while monitoring D-dimer levels and reduction in size and tenderness of the right thigh ecchymosis.

On day 7, the FFP dose was reduced to four units/day, and FFP was discontinued on day 8. By day 9, unfractionated heparin had been reduced to 10 units/kg/hour, and the purpura on the right thigh had nearly resolved by day 10. This reduction in anticoagulation intensity coincided with a temporary improvement in coagulation biomarkers (Table 2).

**Table 2:** Serial laboratory parameters and associated clinical events during hospitalization ^a^ Data on Day 11 were obtained after increasing the heparin dose to 20 units/kg/hour in response to the splenic infarction PT-INR, prothrombin time-international normalized ratio; APTT, activated partial thromboplastin time; FDP, fibrin degradation products; VTE, venous thromboembolism

Hospital Day	PT-INR	APTT (seconds)	D-dimer (µg/mL)	FDP (µg/mL)	Clinical notes
1	2.13	53	1.7	2.6	Admission, diagnosis of VTE
4	1.25	41	0.3	2.5	Improvement under therapy
8	1.71	90.8	0.3	2.5	Purpura resolved
11	2.13	200^a^	28.8	48.7	Onset of splenic infarction
12	2.19	200	51.7	67.2	-
13	2.18	200	14.1	18.8	-
15	2.27	200	3.6	4.7	-
17	2.69	200	1.2	2.5	-
24	4.06	63	0.4	2.5	Heparin discontinued
29	5.76	86.8	0.3	2.5	Discharge
Reference Range	0.9~1.1	25~35	<1.0	<5.0	-

**Figure 2:**
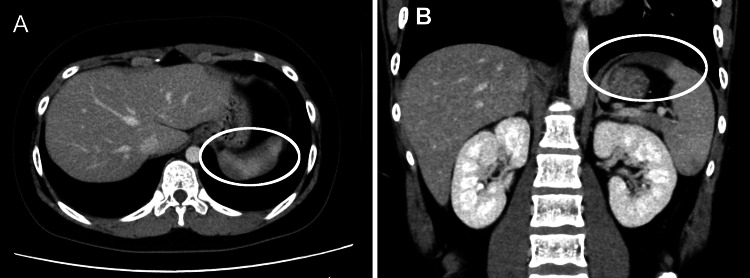
Axial (A) and coronal (B) contrast-enhanced CT images showing splenic infarction A well-demarcated, wedge-shaped hypoenhancing area is seen in the spleen (white circles), consistent with splenic infarction.

While anticoagulation was being tapered, around day 11, the patient suddenly developed abdominal pain. Laboratory tests performed at that time showed a marked increase in D-dimer (28.8 μg/mL) and TAT (59.6 ng/mL), indicating a sudden exacerbation of the coagulation pathway (Table 2). A contrast-enhanced CT scan revealed a partial splenic infarction (Figure 2).

The patient was treated conservatively with intensified anticoagulation, including an increased heparin dose to 20 units/kg/hour and supplementation with FFP at 8 units/day. 

Following the diagnosis of splenic infarction, anticoagulation was intensified. Recombinant human antithrombin (Acoalan; Japan Blood Products Organization, Tokyo, Japan) was administered at 1,800 units/day from days 11 to 20. Beginning on day 16, activated protein C concentrate (Anact C; KM Biologics Co., Ltd., Kumamoto, Japan) was administered for three days during the subacute phase, with dose adjustments detailed in Figure 1. Heparin was gradually tapered thereafter and continued until day 23, while FFP was reduced to 4 units/day from days 12 to 15 and then discontinued. No further thrombotic events occurred after discontinuation of heparin, and the patient was discharged without complications on day 29 of hospitalization. A summary of the clinical course is shown in Figure 1. Follow-up imaging performed on day 39 confirmed resolution of the splenic infarction. Investigations, including echocardiography and pulmonary scintigraphy, ruled out cardiac shunt-related embolism or other systemic causes of thrombosis.

Summary of the clinical timeline

To clarify the temporal relationship between COVID-19 infection and thrombotic events, the key clinical events are summarized as follows: (i) Two days prior to admission: Onset of fever and cough; (ii) Day 1 (Admission): Diagnosis of COVID-19 via antigen testing; onset of right thigh pain and ecchymosis; admission for recurrent VTE; (iii) Days 4-10: Improvement of ecchymosis and normalization of coagulation markers (D-dimer/FDP) under anticoagulant therapy; (iv) Day 11 (13 days post-symptom onset): Sudden onset of abdominal pain; diagnosis of splenic infarction via contrast-enhanced CT; (v) Day 29: Discharged without sequelae.

## Discussion

Severe congenital protein C deficiency poses substantial challenges in anticoagulation management. In this patient, splenic infarction occurred during heparin tapering in the setting of COVID-19, underscoring the difficulty of maintaining adequate anticoagulation in high-risk situations. Protein C deficiency confers an increased risk of both venous and arterial thrombosis, as demonstrated in previous cohort studies [7,8], and the presence of recurrent VTE and severely reduced protein C antigen levels further complicated management in this case. Protein C supplementation was considered necessary at the time of admission. However, because concentrate was initially unavailable, FFP was used as an alternative. Although the initial VTE improved with anticoagulation, the patient subsequently developed a splenic infarction. COVID-19-associated coagulopathy, characterized by endothelial injury and sustained inflammation, may have contributed to an elevated background thrombotic tendency [2,3].

Splenic infarction has been documented during both the acute and recovery phases of COVID-19 [9,10]. In this case, the event occurred 13 days after symptom onset, consistent with reports that arterial thrombosis can arise from persistent or delayed endothelial dysfunction even after clinical symptoms have resolved [2,3]. Notably, the infarction developed during a period when D-dimer and FDP levels had normalized (day 4-10), and the purpura had resolved. Such biochemical improvement may not necessarily indicate true resolution of hypercoagulability, particularly in patients with severe congenital thrombophilia. In this patient, the normalization of fibrinolytic markers likely reflected pharmacologic suppression of thrombin generation by heparin rather than genuine stabilization of the underlying prothrombotic state. Furthermore, echocardiography revealed no evidence of intracardiac shunt, making paradoxical embolism unlikely. Therefore, the splenic infarction in this case was most consistent with in situ arterial thrombosis triggered by COVID-19-related vascular endothelial injury, rather than embolic disease. This mechanism aligns with emerging evidence that COVID-19 can induce localized arterial thrombosis even in patients whose underlying thrombophilia predominantly affects the venous system.

In patients with congenital protein C deficiency who develop COVID-19, endothelial dysfunction and inflammation may persist longer than clinically apparent, creating a vulnerable period in which thrombotic events can occur despite apparent recovery. Although heparin initially controlled coagulation biomarkers, the patient’s intrinsic thrombotic potential was likely influenced by the combined effects of congenital thrombophilia and COVID-19-related endotheliopathy. Consequently, tapering anticoagulation solely on the basis of biomarker normalization may insufficiently reflect ongoing thrombotic risk, potentially allowing residual hypercoagulability to manifest as arterial thrombosis.

This case highlights that in high-risk patients with severe congenital thrombophilia and COVID-19, anticoagulation management, particularly tapering, should not be guided solely by the normalization of biomarkers like D-dimer. Decisions must integrate the clinical context, the known severity of the underlying thrombophilia, and an awareness of the potential for delayed endothelial injury from COVID-19, which may outlast symptomatic and biochemical improvement. These findings underscore the need to develop clearer anticoagulation management strategies, particularly regarding the safe tapering of therapy, for patients with congenital coagulation disorders during and after COVID-19 infection [11]. The lack of imaging confirmation for the initial recurrent VTE, while clinically justified to prioritize rapid intervention, is a minor limitation of this report. The spleen can be a target organ in various hematologic and thrombotic diseases. While infarctions may occur in hypercoagulable states, other severe complications like spontaneous rupture are documented in the context of hematologic malignancies [12]. Reporting such atypical splenic manifestations is therefore essential to enhance clinician awareness, facilitate early recognition, and prevent fatal outcomes.

## Conclusions

This case underscores the elevated risk of arterial thrombosis in patients with protein C deficiency who develop COVID-19, even during periods of apparent biochemical improvement. The occurrence of splenic infarction illustrates the combined effect of congenital thrombophilia and COVID-19-associated endotheliopathy. Close monitoring and carefully tailored anticoagulation strategies are essential to prevent thrombotic complications in such patients during and after COVID-19, regardless of disease severity. Clinicians should exercise caution when tapering anticoagulation based solely on biomarker normalization, as underlying hypercoagulability may persist despite apparent recovery.
